# Infrapatellar fat pad fibrosis after anterior cruciate ligament reconstruction is associated with male sex, high body mass index, prolonged operation time and articular cartilage damage, with detrimental effects on one‐year clinical outcomes

**DOI:** 10.1002/jeo2.70365

**Published:** 2025-07-18

**Authors:** Ryu Yoshida, Hideyuki Koga, Tomomasa Nakamura, Nobutake Ozeki, Mai Katakura, Masaki Amemiya, Takashi Hoshino, Aritoshi Yoshihara, Toyohiro Katsumata, Yasumasa Tokumoto, Ichiro Sekiya, Yusuke Nakagawa

**Affiliations:** ^1^ Department of Joint Surgery and Sports Medicine Institute of Science Tokyo Tokyo Japan; ^2^ Center for Stem Cell and Regenerative Medicine Institute of science Tokyo Tokyo Japan; ^3^ Department of Cartilage Regeneration Institute of science Tokyo Tokyo Japan

**Keywords:** anterior cruciate ligament reconstruction, clinical outcomes, infrapatellar fat pad fibrosis, magnetic resonance imaging, risk factors

## Abstract

**Purpose:**

The aim of this study was to examine the risk factors of infrapatellar fat pad (IFP) fibrosis and the associations between the degree of IFP fibrosis and clinical outcomes in patients who underwent anterior cruciate ligament reconstruction (ACLR).

**Methods:**

A total of 97 patients who underwent primary ACLR using autologous hamstring tendons were divided into the mild fibrosis group (M group) and severe fibrosis group (S group), based on IFP fibrosis scoring (Grades 0–5) on magnetic resonance imaging at 3 months postoperatively. Clinical outcomes at 1 year postoperatively were compared between groups. Univariate logistic regression analysis was performed to determine factors associated with IFP fibrosis. Additionally, multiple linear regression analysis was performed to investigate whether IFP fibrosis affected clinical outcomes at 1 year postoperatively.

**Results:**

Patients were classified into the S group (*n* = 21) and the M group (*n* = 76). There were significantly more males (*p* = 0.036), higher body mass index (*p* = 0.004), longer operation times (*p* = 0.031), and more cartilage injuries identified during arthroscopy (*p* = 0.030) in the S than M group. International Knee Documentation Committee (IKDC) subjective scores (*p* = 0.040), and Knee Injury and Osteoarthritis Outcome Score (KOOS) symptoms (*p* = 0.009) and quality of life values (*p* = 0.026) were significantly lower in the S than M group. The range of motion was significantly worse on both extension (*p* < 0.001) and flexion (*p* = 0.002) in the S than M group. Multiple regression analysis revealed IFP fibrosis as an independent factor affecting the IKDC subjective score (*p* = 0.037), KOOS‐symptom subscore (*p* = 0.037) and extension angle (*p* = 0.002).

**Conclusions:**

Male sex, high BMI, prolonged surgery, and articular cartilage damage are risk factors for IFP fibrosis after ACLR. IFP fibrosis affects the range of motion and subjective patient evaluations at 1 year postoperatively. MRI‐based evaluation at 3 months may help identify high‐risk patients, and early interventions targeting fibrosis could improve postoperative recovery.

**Level of Evidence:**

Level III, case–control study.

**Abbreviations**
ACL: anterior cruciate ligament
ACLR: anterior cruciate ligament reconstruction
AKP: anterior knee pain
AM: anteromedial
BMI: body mass index
ICRS: International Cartilage Repair Society Scale
IFP: infrapatellar fat pad
IKDC: International Knee Documentation Committee
KiRA: Kinematic Rapid Assessment
KOOS: Knee injury and Osteoarthritis Outcome Score
LCL: lateral collateral ligament
LM: lateral meniscus
MCID: minimal clinically important difference
MCL: medial collateral ligament
MM: medial meniscus
PCL: posterior cruciate ligament
PM: posterolateral
QOL: quality of life
ROM: range of motion
TNF: tumour necrosis factor

## INTRODUCTION

Anterior cruciate ligament (ACL) injuries are the most frequent sports injuries requiring surgery, and their incidence is increasing [15]. Postoperative complications after ACL reconstruction (ACLR) may lead to intra‐articular fibrosis, which can result in persistent pain and delayed functional recovery [12, 17].

The infrapatellar fat pad (IFP) comprises adipose tissue situated within the knee joint compartment, between the patellar ligament and intra‐articular space. It is located in the intracapsular region of the knee joint but outside the synovial lining [14, 25]. The fundamental role of the IFP is to provide physical cushioning. Belluzzi et al. proposed that the IFP bridges gaps in the joint, providing stability to the patella during physical activity and shielding the knee joint from further mechanical damage [3]. Furthermore, studies have indicated that the IFP plays a central nociceptive role and is rich in substance P nerves, indicating that it may serve as a source of pain [5, 11].

Anterior knee pain (AKP) following ACLR is associated with delayed muscle recovery and unfavourable clinical outcomes [8]. One significant contributing factor to AKP is IFP pathology, such as Hoffa disease (IFP impingement) or inflammation [14, 18]. Previous research suggests that IFP fibrosis is associated with AKP following ACLR [28]. Additionally, IFP fibrosis reduction has been reported to alleviate pain, indicating a link between IFP fibrosis and knee pain [1, 36]. Further, recent research by Onuma et al. indicates that chronic IFP fibrosis leads to residual pain in a rat model of IFP fibrosis [33]. As such, the presence of IFP fibrosis could contribute to negative clinical outcomes. However, data on the impact of early postoperative IFP fibrosis on short‐term clinical outcomes in patients with ACLR is lacking.

Therefore, this study aimed to examine the risk factors of IFP fibrosis and associations between the degree of IFP fibrosis and clinical outcomes in patients with ACLR. We hypothesised that a greater degree of IFP fibrosis as assessed by MRI at 3 months after ACLR would have detrimental effects on clinical outcomes, and that longer operation time, additional intra‐articular procedures such as meniscal repair or partial meniscectomy, and cartilage damage would be relevant to IFP fibrosis. Identifying risk factors for IFP fibrosis may help clinicians to identify high‐risk patients, optimise surgical techniques, and develop targeted rehabilitation protocols to minimise the risk of this complication and improve patient outcomes.

## MATERIALS AND METHODS

### Patients

This retrospective study was approved by the Institutional Review Board of Tokyo Medical and Dental University Hospital (research protocol identification number: 1146). Patients who underwent primary double‐bundle ACLR with an autologous semitendinosus tendon between 2014 and 2019 and provided consent to participate in the study prior to ACLR surgery were eligible for inclusion. Patients who underwent revision ACLR, multiple ligament reconstruction, or had bone‐patellar tendon‐bone graft use, prior injuries, or contralateral knee surgeries were excluded. A total of 340 patients were initially screened, and 97 met these criteria and were included in the study.

### Time from injury to surgery

Although the exact date of injury was not recalled by some patients, all were able to report the timing within a range of several days. Accordingly, patients were classified into two groups based on the time from injury to surgery: within 3 months and more than 3 months, in accordance with previously reported thresholds [24].

### Operative procedures

All procedures were performed by the same group of surgeons, consisting of 11 orthopaedic surgeons who each had more than 10 years of experience and received a training of knee surgery for at least two years. First, a conventional arthroscopic procedure was performed using anterolateral and anteromedial portals to verify the ACL tear and assess the condition of the menisci and articular cartilage. The management of meniscal injuries was based on the extent and severity of the injury. ACLR was performed using the remnant‐preserved anatomic double‐bundle technique with semitendinosus tendon [27]. The autologous semitendinosus tendon was cut and folded to produce two double‐stranded bundles for the creation of anteromedial (AM) and posterolateral (PL) bundles. Femoral and tibial tunnels were created at the anatomical sites of the insertion of each bundle [30]. The femoral tunnel was created using an outside‐in, transportal, or transtibial approach, whereas the tibial tunnel was formed from the AM surface of the tibia. Both AM and PL grafts were inserted through the tibial tunnel into the femoral tunnel, and the femoral portions of both grafts were secured with EndoButton‐CL (Smith and Nephew, Andover, FL, USA). The AM bundle graft was anchored to a staple anchor (Meira Corporation, Aichi, Japan) using sutures at the tibial site, with the initial tension adjusted to be equal per cross‐sectional area on a basis of 25 N per 6 mm in diameter at 20° knee flexion [23]. The PL graft was then secured to another anchor staple using a similar procedure.

### Postoperative management

A standardised postoperative rehabilitation protocol was uniformly applied to all patients, with the exception that those who underwent meniscal repair were prohibited from bearing weight beyond 90° of flexion for 3 months postoperatively. Range of motion (ROM) exercises were initiated on the day following the procedure, and patients were permitted to perform walking exercises while wearing a knee immobiliser and relying on crutches for 4 weeks. Patients who regained more than 65% of their muscle strength compared to that of the uninjured side were allowed to start running exercises at 3 months postoperatively. Patients who achieved > 90% recovery in muscle strength were permitted full athletic activity at 6 months postoperatively.

### Evaluation at the time of surgery

Data on the operative time, presence of cartilage damage, and meniscus treatment were collected from surgical records. Cartilage damage was assessed on a 5‐point scale using the International Cartilage Repair Society Scale (ICRS) as follows: Grade 0, normal; Grade 1, nearly normal; Grade 2, lesion of < 50% thickness of the cartilage depth; Grade 3, lesion of > 50% thickness of the cartilage depth, without violation of the subchondral bone plate; and Grade 4, lesion extending through the subchondral bone plate [7], with Grades 0–1 considered as reflecting no cartilage damage and Grades 2–4 reflecting cartilage damage. Meniscus treatment was considered as present if a procedure was performed, regardless of whether it was sutured or resected.

### Magnetic resonance imaging (MRI) evaluations

**Figure 1:**
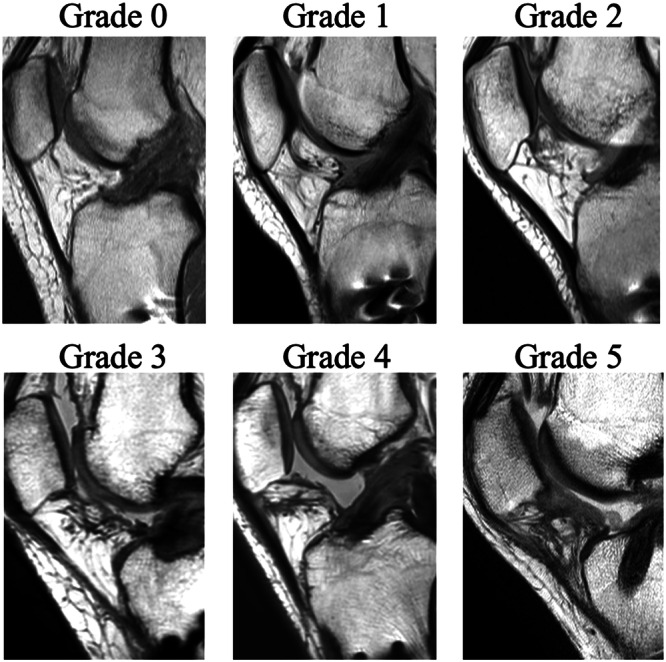
Proton density sagittal magnetic resonance image (MRI) classifying IFP fibrosis from Grade 0 to Grade 5. The definitions are as follows; Grade 0, no fibrosis; Grade 1, focal fibrosis; Grade 2, between Grades 1 and 3; Grade 3, complete band fibrosis from the lower pole of the patella to the tibial plateau; Grade 4, between Grades 3 and 5; Grade 5, diffuse and infiltrated fibrosis (occupying more than 75% of the area).

MRI was performed in a non‐weight‐bearing supine position at 3 months postoperatively. Proton‐weighted images in the sagittal view of the reconstructed ACL were used. IFP fibrosis was classified into six grades based on fibrosis patterns (Figure 1), as follows: Grade 0, no fibrosis; Grade 1, focal fibrosis; Grade 2, between Grades 1 and 3; Grade 3, complete band fibrosis from the lower pole of the patella to the tibial plateau; Grade 4, between Grades 3 and 5; Grade 5, diffuse and infiltrated fibrosis (occupying more than 75% of the area) [47]. Specifically, the modified Yoon score was used [29, 47]. Patients were divided into two groups according to IFP fibrosis score: the severe fibrosis group (S group) for Grades 4 and 5, and the mild fibrosis group (M group) for Grades 0–3 [29]. A single investigator who was blinded to the patients' background and clinical outcomes graded the IFP fibrosis severity. To assess the reliability of the grading, 20 cases were randomly selected. Two independent investigators (R.Y. and Y.T), blinded to each other's evaluations, graded these cases to determine inter‐class correlation coefficient (inter‐ICC). In addition, intra‐class correlation coefficient (intra‐ICC) was evaluated by having the same investigator (R.Y.) re‐assess the same 20 cases after an interval of over one year. The inter‐ICC(2,1) was 0.89, and intra‐ICC(2,1) was 0.72. Both indicated good reliability, as it was > 0.7.

### Clinical evaluations

Clinical evaluations were performed at 1 year postoperatively by orthopaedic surgeons certified by the Japanese Orthopaedic Association. The Lysholm knee scale was used for a general evaluation of the knee. Subjective patient satisfaction and sports performance were assessed from 0 to 100 points. The International Knee Documentation Committee (IKDC) Subjective Knee Form and Knee Injury and Osteoarthritis Outcome Score (KOOS) were also used [2, 20, 37]. The knee extension angle was measured in 1° increments and the flexion angle was measured in 5° increments using a goniometer. The maximum extension strength (kg) of both knees was measured using a Cybex machine (Lumex, Ronkonkoma, NY, USA) at 60°/s. The measurement accuracy was one decimal place. The extensor muscle strength index was expressed as a percentage of the strength of the non‐operated knee.

### Statistical analyses

Statistical analyses were performed using STATA 15.0 software (StataCorp, College Station, TX, USA). Patient demographics and clinical outcomes were compared between severe and mild fibrosis groups using the Student's *t*‐test or chi‐square test. Logistic regression analysis was performed to determine the risk factors for IFP fibrosis. Furthermore, multiple linear regression analyses were conducted to assess the strength of the relationship between clinical outcomes at 1 year postoperatively and eight predictor variables, including IFP fibrosis, patient demographics such as age, sex, body mass index (BMI), preoperative Tegner activity scale score, and operative findings such as operation time, meniscus treatment, and cartilage damage. For all analyses, *p*‐values < 0.05 were considered as statistically significant.

The required sample size was estimated using using G‐power 3.1 software (Kiel University, Kiel, Germany). Based on a previous study [29], the effect size of the knee extension angle at one year after ACLR was calculated as 0.79. Given the 1:4 ratio of the S and M groups, a total of 82 patients were required as the minimum sample size to obtain a power of 0.80 at an alpha level of 0.05.

## RESULTS

### Patient demographics and intraoperative findings

Ninety‐seven patients were included in this study. There were 21 patients (men: *n* = 12; women: *n* = 9) in the S group and 76 patients (men: *n* = 23; women: *n* = 53) in the M group. There was no difference in age at the time of surgery between the two groups, whereas there were significantly more men in the S group than in the M group (Table 1). In terms of BMI, the M group (25.1 kg/m^2^) was clearly larger than the S group (23.0 kg/m^2^) (Table 1), and there was no significant difference in time from injury to surgery between the two groups (Table 1).

**Table 1:** Patients' demographic data.

	Severe fibrosis (*N* = 21)		Mild fibrosis (*N* = 76)		
	*N* or mean	%, or SD, range	*N* or mean	%, SD or range	*p* value
Age at surgery, years	28.0	9.3, 16‐44	27.2	12.9, 15‐59	n.s
Sex					
Male	12	57.1%	23	30.3%	0.036
Female	9	42.9%	53	69.7%	
BMI kg/m^2^	25.1	3.4	23.0	2.7	0.004
Tegner activity scale	6.9	1.4, 4–10	6.7	1.3, 3–9	n.s
Operation time, min	168.0	65.4, 72–350	135.4	57.7, 68–337	0.031
Meniscus treatment					
Treatment	11	52.4%	24	31.6%	n.s
Non‐treatment	10	47.6%	52	68.4%	
Cartilage injury					
Presence	10	47.6%	58	76.3%	0.030
Absence	11	52.4%	18	23.7%	
Time from injury to surgery					
0–3 months	11	52.4%	40	52.6%	n.s
>3 months	10	47.6%	36	47.4%	

*Note*: *p* value by either Student *t* test or Chi‐square test.

Abbreviations: BMI, body mass index; *N*, numbers; n.s., not significant; *SD*, standard deviation.

Operation times were significantly longer in the S group (168.0 min) than in the M group (135.4 min) (*p* = 0.031). Cartilage damage was more frequently observed in the S group (negative: *n* = 11; positive: *n* = 10) than in the M group (negative: *n* = 58; positive: *n* = 18) (*p* = 0.030). No significant differences were found between the two groups in terms of meniscal treatment (Table 1).

Univariate logistic regression analysis also revealed significant differences between the two groups in male sex (Table 2; odds ratio [OR] = 3.072, *p* = 0.027), BMI (OR = 1.268, *p* = 0.007), operation time (OR = 1.008, *p* = 0.038) and cartilage damage (OR = 3.102, *p* = 0.028).

**Table 2:** Univariate logistic regression analysis.

	OR	95%CI	*p* value
Age at surgery	1.006	0.967–1.046	n.s
Sex (Male)	3.072	1.138–8.295	0.027
BMI	1.268	1.068–1.504	0.007
Preoperative Tegner activity scale	1.141	0.770–1.691	n.s
Operation time (min)	1.008	1.000–1.016	0.038
Meniscus treatment (presence)	0.508	0.190–1.357	n.s
Cartilage damages (presence)	3.102	1.127–8.539	0.028

Abbreviations: CI, confidence interval; BMI, body mass index; OR, odds ratio; n.s., not significant.

### Clinical outcomes

The clinical outcomes at one year postoperatively are shown in Table 3. There were significant differences between the S and M groups in the IKDC subjective score (83.1 vs. 88.5, *p* = 0.040) and KOOS‐symptom (87.2 vs. 93.4, *p* = 0.009), and KOOS‐quality of life (QOL) subscores (76.8 vs. 86.2, *p* = 0.026). Patients in the S group had greater extension and flexion angle restrictions than those in the M group (extension: 0.0° vs. 1.8°, *p* < 0.001; flexion: 145.9° vs. 150.4°, *p* = 0.002). Patients in the S group tended to have lower knee extension muscle strength than those in the M group (extension: 88.8% vs. 96.7%, *p* = 0.08). Multiple regression analysis revealed IFP fibrosis as an independent factor affecting the IKDC subjective score (*β* = −6.05, *p* = 0.037), KOOS‐symptom subscore (*β* = −5.24, *p* = 0.037), and extension angle (*β* = −1.40, *p* = 0.002) (Supporting Information: Table 1).

**Table 3:** Clinical outcomes 1 year after surgery.

	Severe fibrosis (*N* = 21)		Mild fibrosis (*N* = 76)		
	*N* or mean	%, SD	*N* or mean	%, SD	*p* value
Lysholm score	94.8	5.2	96.7	4.8	n.s
IKDC score	83.1	10.2	88.5	10.5	0.040
KOOS					
Symptom	87.2	9.9	93.4	9.0	0.009
Pain	93.3	7.0	94.4	7.0	n.s
ADL	99.1	1.6	98.6	3.3	n.s
Sports/rec	86.9	11.8	88.7	12.6	n.s
QOL	76.8	20.0	86.2	15.5	0.026
Subjective evaluation, %	90.5	16.7	90.0	13.8	n.s
Knee extension angle	0.0	1.6	1.8	1.5	<0.001
Knee flexion angle	145.9	7.3	150.4	5.2	0.002
Extension muscle strength					
Index, % (operated/healthy side)	88.8	16.8	96.7	17.9	n.s
Tegner activity scale	6.7	1.5	6.5	1.4	n.s

*Note*: *p* value by student *t*‐test.

Abbreviations: ADL, activity daily life; IKDC, International Knee Documentation Committee; KOOS, Knee injury and Osteoarthritis Outcome Score; *N*, numbers; n.s., not significant; QOL, quality of life; rec, recreation; SD, standard deviation.

## DISCUSSION

The most important findings of this study were that IFP fibrosis at 3 months post‐ACLR surgery was associated with male sex, high BMI, longer operation time, and articular cartilage damage, and led to unfavourable clinical outcomes at 1 year postoperatively.

The IFP has several functions. The IFP provides vascular supply to the ACL and partly nourishes the ACL via several arteries that feed into the IFP [6, 10]. Additionally, the IFP acts as a physical cushion, protecting the knee joint from mechanical damage [3, 45]. The pressure and volume of the IFP constantly change during exercise and it acts as a cushion between the anterior tibial plateau and patellar tendon [5]. IFP resection can lead to increased intraarticular patellofemoral pressure [34]. In addition, IFP can be a source of pain. Dye et al. arthroscopically assessed the intensity of knee pain and found that the synovium and fat had higher pain ratings than the cruciate ligaments and articular cartilage, while the IFP showed the lowest pain threshold in the knee joint [11].

AKP after ACLR may cause delayed muscle recovery, leading to poor clinical outcomes. IFP fibrosis is considered to be involved in AKP after ACLR [28, 31, 47]. Additionally, IFP fibrosis is associated with osteoarthritic changes in patellofemoral joints [47]. Nakagawa et al. reported that severe IFP fibrosis at 3 months after ACLR was associated with elevated inflammatory cytokine levels in the synovial fluid at 3 or 4 days postoperatively, leading to poor short‐term clinical outcomes after ACLR [29]. In the present study, the S group had significantly worse outcomes than the M group in terms of the ROM, IKDC score, and KOOS‐symptom and KOOS‐QOL subscores at 1 year postoperatively, with a trend towards worse results in other evaluations. Although KOOS‐pain subscores did not significantly differ between the S and M groups, the S group showed significantly worse KOOS‐symptom and KOOS‐QOL subscores. Previous studies have reported the minimal clinically important difference (MCID) to be 11.5 points for the IKDC score, and approximately 8–10 points for the KOOS [21, 38, 46]. While the between‐group difference in the IKDC score did not reach the MCID, the differences in KOOS‐Symptom and KOOS‐QOL were close to their respective thresholds, with KOOS‐Symptom in particular exceeding the MCID. Futhermore, IFP fibrosis was identified as an independent factor to affect to KOOS‐symptom. These results indicated that IFP fibrosis at 3 months postoperatively may lead to knee symptoms, such as a catching sensation, swelling, and stiffness, at 1 year after ACLR.

According to previous studies, acute intraarticular inflammation can be a factor in the pathogenesis of IFP fibrosis after ACLR, and the synovial tissue around the knee (especially the IFP) produces and stores inflammatory cytokines [17, 29, 43]. In a rat model of knee arthritis induced by intra‐articular injection of monoiodoacetic acid, extensive fibrotic changes in the IFP occurred, along with the destruction of articular cartilage [19]. Degeneration of the patellofemoral articular cartilage has also been reported to cause pain after ACLR [16]. In the present study, IFP fibrosis severity grade was associated with longer operative time and the presence of cartilage damage, suggesting that IFP fibrosis is associated with operative invasiveness and intraarticular damage.

Currently, there is no consensus on whether sex is associated with arthrofibrosis, such as IFP fibrosis, after ACLR [9, 32, 39]. Cosgarea et al. reported that sex was not an independent risk factor of arthrofibrosis after ACLR [9], while Nwachukwu et al. reported that female sex was a risk facotor of arthrofibrosis [32]. Furthermore, Sanders et al. reported that risk of arthrofibrosis was 2.5 times higher in women than in men [39]. In contrast, in the present study, IFP fibrosis was more common in men than in women, suggesting that IFP fibrosis may differ from arthrofibrosis with respect to the underlying pathological conditions.

A longer time from injury to surgery has been reported as a risk factor for IFP fibrosis [4, 42]. Bierke et al. found that patients who underwent ACLR more than 21 days after injury had a significantly higher incidence of arthrofibrosis (15.2%) compared to those treated within 21 days (5.6%) (*p* = 0.01), emphasising the importance of early surgical intervention [4]. In the present study, we examined whether ACLR was performed within three months of injury, based on previous literature [24]. However, in our analysis, the timing of surgery did not appear to be a contributing factor to the severity of IFP fibrosis.

Logistic regression analysis showed that BMI at surgery was one of risk factor of IFP fibrosis at 3 months post‐ACLR. Inside the IFP, two primary origins of inflammatory mediators exist: adipocytes, and resident and infiltrating leucocytes, mainly macrophages and lymphocytes [40]. Jedrzejczyk et al. reported from a study of 74 human IFPs that there was an increase in the numbers of adipocytes and infiltrating lymphocytes in the IFP of patients with higher BMI [22]. In addition, increased adipocyte numbers in the obese IFP have been shown to contribute to increased levels of cytokines, adipokines and growth factors such as TNFα, IL‐6, leptin, vascular endothelial growth factor and basic fibroblast growth factor in the knee joint [35, 44]. These results suggested that IFP in high BMI patients induced more inflammatory cytokines and intensifies joint inflammation, which may have resulted in more severe IFP fibrosis. Clinically, although BMI was not an independent factor to affect clinical outcomes in this study, the Moon knee group with a larger cohort study of 1592 ACLR patients revealed that a high BMI was a detrimental factor for10‐year clinical outcomes after ACLR [16]. Surgeons may need to consider postoperative IFP fibrosis when performing ACLR in patients with a high BMI.

Multiple regression analysis revealed that IFP fibrosis at 3 months post‐ACLR was a risk factor for knee extension limitations at 1 year postoperatively. Shelbourne et al. defined more than 2° of extension loss compared to the contralateral knee as a clinically meaningful difference after ACLR [41]. In the present study, the between‐group difference in knee extension angle was 1.8°, which did not exceed this threshold but was close to it. Mayr et al. reported that preoperative knee joint extension restriction and perioperative pain may lead to arthrofibrosis [26]. Further, Fisher and Shelbourne reported that approximately 4% of patients require arthroscopic intervention because of knee joint extension limitations caused by arthrofibrosis at approximately 9 months after ACLR [13]. These findings suggest that a more cautious rehabilitation approach is required in patients with IFP fibrosis to prevent extension restriction.

This study had some limitations. First, although the number of patients was sufficient, as indicated by power analysis, the patient cohorts were relatively small; only 21 patients were included in the S group. Accordingly, multiple logistic regression analysis could not be performed to determine the risk factors for severe IFP fibrosis after adjusting for confounding factors. Further research with a larger number of cases is necessary to validate the obtained results and perform additional statistical analyses. Second, there was an imbalance in the number of patients between the two groups, which may increase the risk of statistical errors. However, severe IFP fibrosis after ACLR is relatively uncommon, and this distribution reflects the actual clinical prevalence, making the imbalance difficult to avoid. Third, there was variation in the experience of the physicians making physical assessments, which may have affected the results of the knee ROM and extensor muscle strength index. Another limitation of this study is the relatively short follow‐up period of one year. This duration may not be sufficient to fully assess the long‐term effects of IFP fibrosis, including its potential association with the development of osteoarthritis and the durability of ACLR outcomes.

Despite the above‐mentioned limitations, the current study results have important clinical implications. Namely, IFP fibrosis should be considered as a predictor of slow recovery and worse short‐term subjective outcomes in patients who undergo ACLR. Surgeons should take care to avoid IFP damage and prolonged operation time during ACLR surgery, especially when operating on male patients with cartilage injuries. Furthermore, the evaluation of IFP fibrosis severity using MRI at 3 months postoperatively may help identify high‐risk patients who are more likely to experience poor functional recovery. Severe IFP fibrosis should be recognised as a potentially harmful postoperative complication that requires clinical attention, particularly in patients with multiple risk factors, as identified in the present study. In such cases, early intervention—including intra‐articular hyaluronic acid (HA) injections—may be worth considering to reduce fibrosis and improve outcomes, as supported by recent preclinical findings [36].

## CONCLUSIONS

IFP fibrosis at 3 months after ACLR affects patient‐reported outcomes at 1 year postoperatively. IFP fibrosis can result in poor recovery in the ROM and extensor muscle strength. Male sex, high BMI, prolonged operation time, and cartilage damage are associated with IFP fibrosis on MRI at 3 months after ACLR, suggesting that early MRI evaluation may help identify high‐risk patients. In such cases, interventions targeting fibrosis could be considered to improve clinical outcomes.

## AUTHOR CONTRIBUTIONS

Ryu Yoshida interpreted MRIs, managed data, performed statistical analysis, participated in study design and wrote the manuscript. Hideyuki Koga acquisition of data, participated in study design. Tomomasa Nakamura, Nobutake Ozeki, Mai Katakura, Masaki Amemiya, Takashi Hoshino, Aritoshi Yoshihara, Toyohiro Katsumata, Yasumasa Tokumoto and Ichiro Sekiya acquisition of data. Yusuke Nakagawa acquisition of data, participated in study design, interpreted results, edited the manuscript and had full access to all of the data in the study and final approved manuscript. All authors read, approved the final manuscript and takes responsibility for the integrity of the data and the accuracy of the data analysis.

## CONFLICT OF INTEREST STATEMENT

The authors declare no conflicts of interest.

## ETHICS STATEMENT

This study was approved by the Institutional Review Board in Institute of science Tokyo (research protocol identification number: 1146). All study participants provided their full written informed consent for participation in this clinical research prior to the operative procedure.

## Supporting information

Supp Table 1.

## Data Availability

The data sets generated and/or analysed during the current study are not publicly available due to the presence of personally identifiable information but are available from the corresponding author upon reasonable request.
