# Correction to “Infrapatellar fat pad fibrosis after anterior cruciate ligament reconstruction is associated with male sex, high body mass index, prolonged operation time and articular cartilage damage, with detrimental effects on one‐year clinical outcome

**DOI:** 10.1002/jeo2.70747

**Published:** 2026-05-15

Yoshida R, Koga H, Nakamura T, Ozeki N, Katakura M, Amemiya M, et al. Infrapatellar fat pad fibrosis after anterior cruciate ligament reconstruction is associated with male sex, high body mass index, prolonged operation time and articular cartilage damage, with detrimental effects on one‐year clinical outcomes. *J Exp Orthop*. 2025;12(3):e70365. https://doi.org/10.1002/jeo2.70365

In Table 1, the values for cartilage injury in the mild fibrosis group were incorrectly presented. The values for ‘presence’ and ‘absence’ were inadvertently reversed. The correct values are as follows: presence (*n* = 18, 23.7%) and absence (*n* = 58, 76.3%). The results reported in the text remain correct, and no other changes are required.

**Table 1:** Patients' demographic data.

	Severe fibrosis (*N* = 21)		Mild fibrosis (*N* = 76)		
	*N* or mean	%, or SD, range	*N* or mean	%, SD or range	*p*‐value
Age at surgery, years	28.0	9.3, 16–44	27.2	12.9, 15–59	n.s
Sex					
Male	12	57.1%	23	30.3%	0.036
Female	9	42.9%	53	69.7%	
BMI kg/m^2^	25.1	3.4	23.0	2.7	0.004
Tegner activity scale	6.9	1.4, 4–10	6.7	1.3, 3–9	n.s
Operation time, minutes	168.0	65.4, 72–350	135.4	57.7, 68–337	0.031
Meniscus treatment					
Treatment	11	52.4%	24	31.6%	n.s
Non‐treatment	10	47.6%	52	68.4%	
Cartilage injury					
Presence	10	47.6%	18	23.7%	0.030
Absence	11	52.4%	58	76.3%	
Time from injury to surgery					
0–3 months	11	52.4%	40	52.6%	0.98
>3 months	10	47.6%	36	47.4%	

*Note*: *p*‐value by either Student *t*‐test or chi‐square test.

Abbreviations: BMI, body mass index; *N*, numbers; n.s., not significant; SD, standard deviation.

A corrected version of Table 1 appears below.

We apologize for this error.

