# Supplementary material for: Infrapatellar fat pad fibrosis after anterior cruciate ligament reconstruction is associated with male sex, high body mass index, prolonged operation time and articular cartilage damage, with detrimental effects on one‐year clinical outcomes
Source: J Exp Orthop. 2025 Jul 18;12(3):e70365. doi: 10.1002/jeo2.70365 (PMC12272509; doi:10.1002/jeo2.70365)
Supplement: Supplementary file 1 — Supp Table 1. [file JEO2-12-e70365-s001.docx]

Supplemental Table. 1 Multiple regression analyses of clinical outcomes 1 year postoperatively

|  |  |  | KOOS | | | | |
| --- | --- | --- | --- | --- | --- | --- | --- |
|  | Lysholm | IKDC | Symptom | Pain | ADL | Sports/rec | QOL |
| IFP fibrosis |  | -6.05, P=0.037 | -5.24, P=0.037 | |  |  |  |
|  |  | -11.74 - -0.60 | -10.17 - -0.31 |  |  |  |  |
| Age | -0.11, P=0.024 | -0.40, P=<0.001 | -0.29, P=0.001 | -0.22, P=0.001 | -0.14, P<0.001 | -0.35, P=0.006 |  |
|  | -0.20 - -0.01 | -0.59 - -0.20 | -0.46 - -0.12 | -0.36 - -0.09 | -0.19 - -0.08 | -0.60 - -0.10 |  |
| Sex |  |  |  |  |  |  |  |
|  |  |  |  |  |  |  |  |
| BMI |  |  |  |  |  |  |  |
|  |  |  |  |  |  |  |  |
| Tegner activity scale |  |  |  |  |  |  |  |
|  |  |  |  |  |  |  |  |
| Operation time |  |  |  |  |  |  |  |
|  |  |  |  |  |  |  |  |
| Meniscus treatment |  |  |  |  |  |  |  |
|  |  |  |  |  |  |  |  |
| Cartilage injury |  |  |  |  |  |  |  |
|  |  |  |  |  |  |  |  |

|  | Subjective evaluation, % | Knee extension angle | Knee flexion angle | Extension muscle strength index | Postoperative tegner activity scale |
| --- | --- | --- | --- | --- | --- |
| IFP fibrosis |  | -1.40, P=0.002 |  |  |  |
|  |  | -2.27 - -0.51 |  |  |  |
| Age |  |  | -0.14, P=0.008 |  | -0.03, P<0.001 |
|  |  |  | -0.24 - -0.04 |  | -0.04 - -0.01 |
| Sex |  |  |  |  |  |
|  |  |  |  |  |  |
| BMI |  |  |  |  |  |
|  |  |  |  |  |  |
| Tegner activity scale |  |  |  |  | 0.93, P<0.001 |
|  |  |  |  |  | 0.83 - 1.04 |
| Operation time |  |  |  |  |  |
|  |  |  |  |  |  |
| Meniscus treatment |  |  |  |  |  |
|  |  |  |  |  |  |
| Cartilage injury |  |  |  |  |  |
|  |  |  |  |  |  |

Regression coefficient, p value and 95% confidence interval

Abbreviations: International Knee Documentation Committee; IKDC, Knee injury and Osteoarthritis Outcome Score; KOOS, activity daily life; ADL, recreation; rec, quality of life; QOL.
